# Supplementary material for: NOTCH1-Related Leukoencephalopathy: A Novel Variant and Literature Review
Source: Int J Mol Sci. 2024 Mar 1;25(5):2864. doi: 10.3390/ijms25052864 (PMC10932310; doi:10.3390/ijms25052864)
Supplement: Supplementary file 1 [file ijms-25-02864-s001.zip › ijms-2863405-supplementary.pdf]

Supplementary Table S1. Neuroradiological features of *NOTCH1* patients including in the review.

Ref	Pt	Age at onset	Age at last contact	Neuroimaging	Variant (NM_017617.5)
**This study**	1	6yr	15yr	Leukoencephalopathy with calcifications and microcysts, TCC, enhancement in the PV white matter, in the intracisternal tract of the trigeminal nerve, and in the meatal tract of the acoustic nerve after gadolinium administration. Decreased NAA and Cho peaks in the PV white matter on MRS. MRI follow-up four years later, showed only a reduction of enhancement after gadolinium.	c.4788_4799dup, p.(Leu1600_His1601insSerArgValLeu)
**Nicita et al.**	1	1mo	2yr	Brain MRI performed at age 8 months showed leukoencephalopathy, calcifications and cysts, enlarged lateral ventricles and bilateral frontotemporal subarachnoid spaces, and TCC. MRI follow-up at age 1.5 years was unchanged (not shown).	c.4811 T > G, p.(Val1604Gly)
**Helman et al.**	1	1 yr	25*	Brain MRI ranged from 11 months to 57 years. All MRIs demonstrated periventricular and deep cerebral white matter signal abnormalities, increasing over time. MRI and CT showed numerous white matter calcium deposits, larger in patients 1, 2, 3, and 4, more numerous and smaller in patients 5, 6, and 7. All patients had subtle brain atrophy, most pronounced in patients 6 and 7.	c.4814_4819dupTCTTCA p.(Phe1606_Lys1607insIlePhe)
	2	15 yr	37*		c.5046C>G p.(Asn1682Lys)
	3	40 yr	65 yr		c.4583G>A p.(Cys1528Tyr)
	4	1 yr	28 yr		c.5046_5047delCCInsAG p.(Asn1682_Arg1683insLysGly)
	5	0 yr	9 yr		c.5042A>G p.(Asp1681Gly)

	6	0 yr	6 yr		c.5078T>C<br>p.(Phe1693Ser)
	7	0 yr	6 yr		c.4754T>C<br>p.(Leu1585Pro)

Mo: month/months. Y: year/years. MRI: magnetic resonance imaging. TC: computed tomography. MRS: magnetic resonance spectroscopy. TCC: thin corpus callosum. PV: periventricular. NAA: N -acetylaspartate. Cho: Choline.
